# Identification of PADI2 as a potential breast cancer biomarker and therapeutic target

**DOI:** 10.1186/1471-2407-12-500

**Published:** 2012-10-30

**Authors:** John L McElwee, Sunish Mohanan, Obi L Griffith, Heike C Breuer, Lynne J Anguish, Brian D Cherrington, Ashley M Palmer, Louise R Howe, Venkataraman Subramanian, Corey P Causey, Paul R Thompson, Joe W Gray, Scott A Coonrod

**Affiliations:** 1 Baker Institute for Animal Health, College of Veterinary Medicine, Cornell University, 122 Hungerford Hill Road, Ithaca, NY, 14853, USA; 2 Lawrence Berkeley National Laboratory, Life Sciences Division, Cancer and DNA Damage Responses, One Cyclotron Road, Berkeley, CA, 94720, USA; 3 Department of Zoology and Physiology, University of Wyoming, 1000 E. University Ave, Laramie, WY, 82071, USA; 4 Department of Cell and Developmental Biology, Weill Cornell Medical College, Box 60, 1300 York Avenue, New York, NY, 10065, USA; 5 Department of Chemistry, The Scripps Research Institute, Scripps Florida, 120 Scripps Way, Jupiter, FL, 33458, USA; 6 Department of Chemistry & Biochemistry, University of South Carolina, 631 Sumter Street, Columbia, SC, 29208, USA; 7 Department of Biomedical Engineering, Oregon Health and Science University, 3303 SW Bond Ave, Portland, OR 97239, USA

**Keywords:** Peptidylarginine deiminase, PAD2/PADI2, HER2/ERBB2, Breast cancer, Luminal, Cl-amidine, Citrullination

## Abstract

**Background:**

We have recently reported that the expression of peptidylarginine deiminase 2 (PADI2) is regulated by EGF in mammary cancer cells and appears to play a role in the proliferation of normal mammary epithelium; however, the role of PADI2 in the pathogenesis of human breast cancer has yet to be investigated. Thus, the goals of this study were to examine whether PADI2 plays a role in mammary tumor progression, and whether the inhibition of PADI activity has anti-tumor effects.

**Methods:**

RNA-seq data from a collection of 57 breast cancer cell lines was queried for PADI2 levels, and correlations with known subtype and HER2/ERBB2 status were evaluated. To examine PADI2 expression levels during breast cancer progression, the cell lines from the MCF10AT model were used. The efficacy of the PADI inhibitor, Cl-amidine, was tested *in vitro* using MCF10DCIS cells grown in 2D-monolayers and 3D-spheroids, and *in vivo* using MCF10DCIS tumor xenografts. Treated MCF10DCIS cells were examined by flow-cytometry to determine the extent of apoptosis and by RT^2^ Profiler PCR Cell Cycle Array to detect alterations in cell cycle associated genes.

**Results:**

We show by RNA-seq that *PADI2* mRNA expression is highly correlated with *HER2/ERBB2* (p = 2.2 × 10^6^) in luminal breast cancer cell lines. Using the MCF10AT model of breast cancer progression, we then demonstrate that PADI2 expression increases during the transition of normal mammary epithelium to fully malignant breast carcinomas, with a strong peak of PADI2 expression and activity being observed in the MCF10DCIS cell line, which models human comedo-DCIS lesions. Next, we show that a PADI inhibitor, Cl-amidine, strongly suppresses the growth of MCF10DCIS monolayers and tumor spheroids in culture. We then carried out preclinical studies in nude (nu/nu) mice and found that Cl-amidine also suppressed the growth of xenografted MCF10DCIS tumors by more than 3-fold. Lastly, we performed cell cycle array analysis of Cl-amidine treated and control MCF10DCIS cells, and found that the PADI inhibitor strongly affects the expression of several cell cycle genes implicated in tumor progression, including *p21*, *GADD45α*, and *Ki67*.

**Conclusion:**

Together, these results suggest that PADI2 may function as an important new biomarker for HER2/ERBB2+ tumors and that Cl-amidine represents a new candidate for breast cancer therapy.

## Background

PADIs are a family of posttranslational modification enzymes that convert positively charged arginine residues on substrate proteins to neutrally charged citrulline, and this activity is alternatively called citrullination or deimination. The PADI enzyme family is thought to have arisen by gene duplication and localizes within the genome to a highly organized cluster at 1p36.13 in humans. At the protein level, each of the five well-conserved PADI members shows a relatively distinct pattern of substrate specificity and tissue distribution [1,2]. Increasingly, the dysregulation of PADI activity is associated with a range of diseases, including rheumatoid arthritis (RA), multiple sclerosis, ulcerative colitis, neural degeneration, COPD, and cancer [3-5]. While the presumptive function of PADI activity in most diseases is linked to inflammation, the role that PADIs play in cancer progression is not clear. We and others, however, have found that PADI4 appears to play a role in gene regulation in cancer cells via histone tail citrullination. For example, in MCF7 breast cancer cells estrogen stimulation enhances PADI4 binding and histone H4 citrullination at the canonical ER target gene, *TFF1,* leading to transcriptional repression [6]. On the other hand, stimulation of MCF7 cells with EGF facilitates activation of *c-fos* via PADI4-mediated citrullination of the ELK1 oncogene [7]. Additionally, others have shown that citrullination of the p53 tumor suppressor protein affects the expression of p53 target genes *p21*, *OKL38*, *CIP1* and *WAF1*[8-10]. Interestingly, treatment of several PADI4-expressing cancer cell lines with the PADI inhibitor, Cl-amidine, elicited strong cytotoxic effects while having no observable effect on non-cancerous lines [11], suggesting that PADIs may represent targets for new cancer therapies.

Our current study suggests that PADI2 may also play a role in cancer progression, and this prediction is supported by several previous studies. For example, a mouse transcriptomics study investigating gene expression in MMTV-neu tumors found that *PADI2* expression was upregulated ~2-fold in hyperplastic, and ~4-fold in primary neu-tumors, when compared to matched normal mammary epithelium [12]. In humans, *PADI2* is one of the most upregulated genes in luminal breast cancer cell lines compared to basal lines [13,14]. Additionally, gene expression profiling of 213 primary breast tumors with known HER2/ERBB2 status identified *PADI2* as one of 29 overexpressed genes in HER2/ERBB2+ tumors; thus, helping to define a HER2/ERBB2+ gene expression signature [15]. Given these previous studies, our goal was to formally test the hypothesis that PADI2 plays a role in mammary tumor progression. For the study, we first documented PADI2 expression and activity during mammary tumor progression, and then investigated the effects of PADI inhibition in cell cultures, tumor spheroids, and preclinical *in vivo* models of breast cancer.

## Methods

### Cell culture and treatment with Cl-amidine

The MCF10AT cell line series (MCF10A, MCF10AT1kC1.2, MCF10DCIS.com, and MCF10CA1aC1.1) was obtained from Dr. Fred Miller (Barbara Ann Karmanos Cancer Institute, Detroit, MI, USA). This biological system has been extensively reviewed [16,17] and culture conditions described [18-20]. The MCF7, BT-474, SK-BR-3, and MDA-MB-231 cell lines were from obtained from ATCC (Manassas, VA, USA) and cultured according to manufacturer’s directions. All cells were maintained in a humidified atmosphere of 5% CO_2_ at 37°C. For the experimental treatment of cell lines with Cl-amidine, cells were seeded in 6-well plates (2 × 10^4^) and collected by trypsinization 5d post-treatment. Counts were performed using a Coulter counter (Beckman Coulter, Fullerton, CA, USA) and are represented as mean fold difference in cell number after treatment. Cl-amidine was synthesized as previously described [21].

### MMTV mice and the generation of MCF10DCIS xenografts and multicellular tumor spheroids

Tissues from the MMTV-neu mouse were a generous gift from Dr. Robert S. Weiss, Cornell University, and the MMTV-*Wnt-1* hyperplastic mammary glands and tumors were a gift of Dr. Louise R. Howe, Weill Cornell Medical College. MCF10DCIS xenograft tumors were generated by injecting 1 × 10^6^ cells in 0.1 mL Matrigel (1:1) (BD Biosciences, San Jose, CA, USA) subcutaneously near the nipple of gland #3 in 6-week old female nude (nu/nu) mice (Taconic, Germantown, NY, USA). When the tumors reached ~200 mm^3^, intraperitoneal injections of Cl-amidine (50 mg/kg/day) or vehicle control (PBS) were initiated and carried out for 14 days. Tumor volume was calculated by the formula: (mm^3^) = (d^2^ × D)/2, where “d” and “D” are the shortest and longest diameters of the tumor, respectively. Tumor volume was measured weekly by digital caliper, and the differences between tumor volumes were evaluated by the non-parametric Mann–Whitney–Wilcoxon (MWW) test. Results are reported as mean ± SD. After 14 days, tumors were removed and either snap-frozen, placed in RNAlater (Qiagen Inc., Valencia, CA, USA), or added to 10% buffered formalin. Seven mice per group were used for each treatment. All mouse experiments were reviewed and approved by the Institutional Animal Care and Use Committees (IACUC) at Cornell University. Multicellular tumor spheroids were generated using the liquid overlay technique as previously described [22-24]. The spheroids were allowed to form over 48h and maintained up to 6–10 days for morphological analysis, then collected, rinsed with phosphate buffered saline, and fixed in 10% buffered formalin.

### Assay of PADI activity

Cell lines were assayed for PADI activity as previously described [25,26]. Briefly, citrulline levels were determined using BAEE (α-N-benzoyl-L-arginine ethyl ester) as a substrate. After incubating lysates for 1h at 50°C with BAEE substrate mixture, the reaction was stopped by the addition of perchloric acid. The perchloric acid-soluble fraction was subjected to a colorimetric reaction with citrulline used as a standard and absorbance measured at 464 nm.

### Immunohistochemistry (IHC) and immunofluorescence (IF)

IHC and IF experiments were carried out using a standard protocol as previously described [27]. Primary antibodies are as follows: anti-PADI2 1:100 (ProteinTech, Chicago, IL, USA), anti-ERBB2 (A0485) 1:100 (Dako, Carpentaria, CA, USA), anti-Cytokeratin 1:100 (Dako), and anti-p63 1:100 (Abcam, Cambridge, MA, USA). Sections prepared for IHC were incubated in DAB chromagen solution (Vector Laboratories, Burlingame, CA, USA) according to the manufacturer’s protocol, washed, and then counterstained with hematoxylin. The IF slides were incubated in streptavidin conjugated-488 (Invitrogen, Carlsbad, CA, USA), washed, and then mounted using Vectashield containing DAPI (Vector Laboratories). Negative controls for both IHC and IF experiments were either rabbit or mouse IgG antibody at the appropriate concentrations. Tumor sections were examined for general morphological differences after hematoxylin and eosin (H&E) staining. Basement membrane integrity was determined using periodic acid-Schiff (PAS) stained slides, and was scored by SM on a scale of 0–3: 0- continuous with no breaching, 1- a few small interruptions, 2- several interruptions with breaching by tumor cells, 3- extensive loss of basement membrane with invasion of tumor cells over the breached area; observations were performed under 10X magnification.

### Immunoblotting

Immunoblotting was carried out as previously described [27]. Primary antibodies were incubated overnight at 4°C using the following concentrations: anti-PADI2 1:1000 (ProteinTech) and anti-ErbB2 1:5000 (Dako). To confirm equal protein loading, membranes were stripped and re-probed with anti-β-actin 1:5000 (Abcam).

### Quantitative real-time PCR (qRT-PCR)

RNA was purified using the Qiagen RNAeasy kit, including on-column DNAse treatment to remove genomic DNA. The resulting RNA was reverse transcribed using the ABI High Capacity RNA to cDNA kit according to the manufacturer’s protocol (Applied Biosystems, Foster City, CA, USA). TaqMan Gene Expression Assays (ABI) for human PADI2 (Hs00247108_m1) and GAPDH (4352934E) were used for qRT-PCR. Data were analyzed by the 2 ^-ΔΔ C(t)^ method [28]. Data are shown as means ± SD from three independent experiments, and were separated using Student’s *t*-test. For the analysis of cell cycle gene expression, cDNA was synthesized (RT^2^ First Strand Kit, Qiagen) and samples analyzed for expression of 84 genes involved in cell cycle regulation by RT^2^ Profiler PCR Cell Cycle Array (PAHS-020A, Qiagen). For data analysis, the RT^2^ Profiler PCR Array software package was used and statistical analyses performed (n = 3). This package uses ΔΔ C_T_–based fold change calculations and the Student’s *t*-test to calculate two-tail, equal variance p-values.

### Flow-cytometry

Monolayers of MCF10DCIS and MCF10A cells were seeded into 25 cm^2^ flasks (2 × 10^6^ cells) and treated with either Cl-amidine (200 μM or 400 μM), or 10μg/mL tunicamycin (apoptosis positive control). BT-474, SK-BR-3, and MDA-MB-231 cell lines were treated as previously described for MCF10DCIS and MCF10A; however, they were also treated with 100 μM Cl-amidine. Cells were harvested after 4d using Accutase (Innovative Cell Technologies, Inc, San Diego, CA, USA), fixed, then permeabilized, and blocked in FACS Buffer (0.1M Dulbecco’s phosphate buffered saline, 0.02% sodium azide, 1.0% bovine serum albumin, and 0.1% Triton X-100) containing 10% normal goat serum and stained (except the isotype controls) with rabbit anti-cleaved Caspase-3 antibody (Cell Signaling Technology, Inc, Danvers, MA, USA). Isotype controls were treated with normal rabbit IgG (Vector Laboratories) at 4 μg/mL. All samples were stained with secondary goat anti-rabbit IgG conjugated to Alexa-488 (Invitrogen) and DAPI (Invitrogen) according to the manufacturer’s instructions. Cells were analyzed on a FACS-Calibur (BD Biosciences) or a Gallios (Beckman Coulter) flow-cytometer and data analyzed for percent apoptotic cells (cleaved Caspase-3+) and cell cycle analysis with FlowJo software (TreeStar, Inc, Ashland, OR, USA). Data are shown as means ± SD from three independent experiments, and were separated using Student’s *t*-test.

### RNA-seq analysis of breast cancer cell lines

Whole transcriptome shotgun sequencing (RNA-seq) was completed on breast cancer cell lines and expression analysis was performed with the ALEXA-seq software package as previously described [29]. Briefly, this approach comprises (i) creation of a database of expression and alternative expression sequence ‘features’ (genes, transcripts, exons, junctions, boundaries, introns, and intergenic sequences) based on Ensembl gene models, (ii) mapping of short paired-end sequence reads to these features, (iii) identification of features that are expressed above background noise while taking into account locus-by-locus noise. RNA-seq data was available for 57 lines (17 basal, 5 basal-NM, 6 claudin-low, 29 luminal). An average of 70.6 million (76bp paired-end) reads passed quality control per sample. Of these, 53.8 million reads mapped to the transcriptome on average, resulting in an average coverage of 48.2 across all known genes. Log2 transformed estimates of gene-level expression were extracted for analysis with corresponding expression status values indicating whether the genes were detected above background level.

### Statistical analysis

All experiments were independently repeated at least three times unless otherwise indicated. Values were expressed as the mean ± the SD. Means were separated using Student’s *t*-test or by Mann—Whitney-Wilcoxon (MWW) test, with a p-value less than 0.05 considered as significantly different. Subtype specific expression in the RNA-seq analysis was determined by Wilcoxon signed-rank test. Correlations were determined by Spearman rank correlation. Genes were considered significantly differentially expressed or correlated if they had a p-value less than 0.05.

## Results

### PADI2 is overexpressed in transformed cells of the MCF10AT model of breast cancer progression

**Figure 1:**
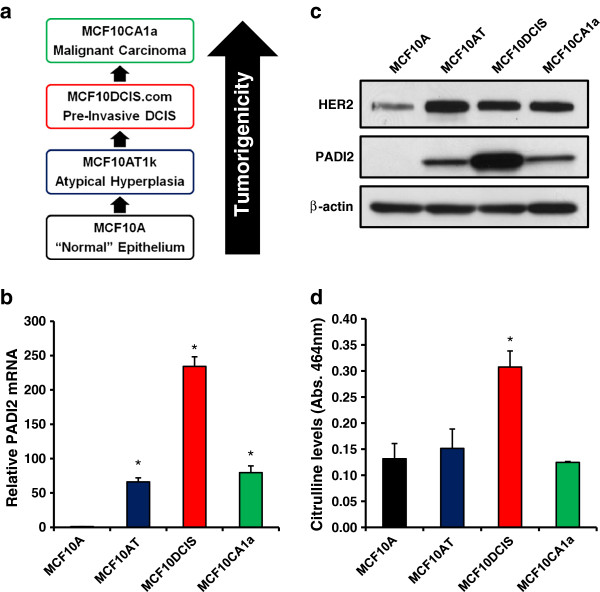
**PADI2 expression is highest in MCF10DCIS.com cells in the MCF10AT model of breast cancer progression. (a)** The MCF10AT model is a series of cell lines that recapitulates the transition from normal epithelium to malignant carcinoma. **(b** and **c)***PADI2* mRNA and protein expression is increased in transformed cells of the MCF10AT model, with very high levels seen in MCF10DCIS cells. Total RNA was isolated and *PADI2* mRNA levels were determined by qRT-PCR (TaqMan) using MCF10A cells as a reference and *GAPDH* normalization. Data were analyzed using the 2 ^-ΔΔ C(t)^ method and are expressed as the mean ± SD from three independent experiments (* p < 0.001). PADI2 expression levels were evaluated by subjecting whole cell lysates to SDS-PAGE and immunoblot analysis using an anti-PADI2 antibody. HER2/ERBB2 expression levels, detected using an anti-HER2 antibody, are also upregulated in the transformed cell lines when compared to the MCF10A levels. The blot was stripped and equal protein loading was determined by probing with a β-actin antibody. (**d**) Citrulline levels in the cell lines were determined by citrullination enzymatic assays, with the highest level of activity measured in the MCF10DCIS cells. Briefly, cell lysates were incubated with PADI substrate BAEE, and the reaction stopped with perchloric acid. The perchloric acid-soluble fraction was subjected to a colorimetric reaction with citrulline used as a standard and absorbance measured at 464 nm.

In order to investigate PADI2 expression during tumor progression, we first utilized TaqMan quantitative real-time PCR (qRT-PCR) to measure *PADI2* mRNA levels in cells from the MCF10AT tumor progression series (Figure 1a). As shown previously, these cell lines closely model the progression from normal (MCF10A), to hyperplastic (MCF10AT), to ductal carcinoma *in situ* (DCIS) with necrosis (MCF10DCIS.com), and finally to invasive/metastatic (MCF10CA1) breast cancer [16,17,30]. Results (Figure 1b) show that *PADI2* mRNA expression is elevated in the transformed cell lines, with the highest levels found in the comedo-DCIS MCF10DCIS.com cell line (hereafter MCF10DCIS). Additionally, PADI2 protein levels closely correlated with *PADI2* mRNA levels across these lines, with the highest levels of PADI2 protein observed in the MCF10DCIS line. Given the previous microarray studies correlating *PADI2* expression with *HER2/ERBB2*, we also probed this cell line series with a well-characterized HER2/ERBB2 antibody and found that HER2/ERBB2 levels were also elevated in the transformed cell lines compared to the non-tumorigenic "normal" MCF10A line (Figure 1c). We also tested whether the increase in PADI2 expression correlated with PADI2 enzymatic activity, with results (Figure 1d) showing that citrulline levels are, in fact, highest in the MCF10DCIS cell line; therefore, indicating a strong correlation between increased PADI2 expression and enzymatic activity. While these cell lines have been previously classified as basal-like [31], both MCF10A and MCF10DCIS have been shown to possess bipotential progenitor properties [19,32,33]. Furthermore, the MCF10AT cells have been reported to show the same multipotent properties [34], but until recently, there has only been one other report showing that HER2/ERBB2 is upregulated in the transformed lines of this series [35]. These data suggest that PADI2 activity may play a role in mammary tumor progression and that PADI2-mediated citrullination may be particularly relevant to comedo-DCIS biology.

### Levels of PADI2 correlate with the luminal breast cancer subtype and HER2/ERBB2 overexpression

**Figure 2:**
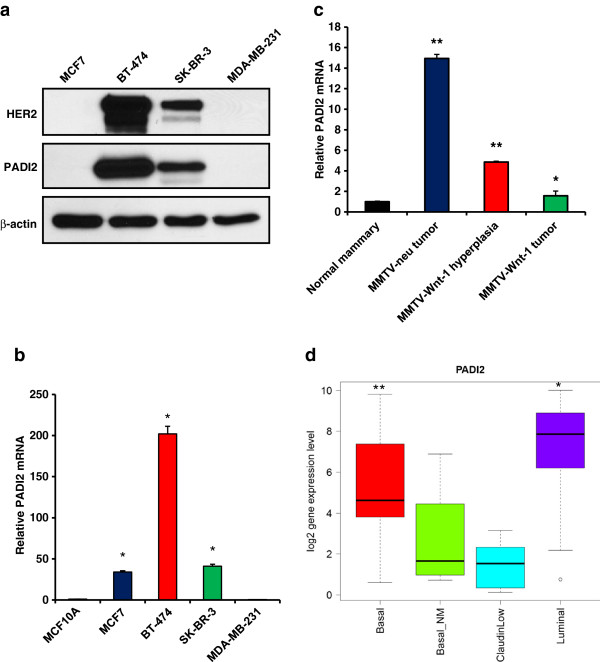
**PADI2 expression is elevated in luminal B BT-474 cells, murine MMTV-neu tumors, and is correlated with the luminal subtype. (a)** Four different breast cancer cell lines were selected to represent the common subtypes of breast cancer: MCF7 (luminal A), BT-474 (luminal B), SK-BR-3 (HER2/ERBB2+), and MDA-MD-231 (basal). PADI2 expression is highest in both of the HER2/ERBB2 overexpressing cell lines, with the highest level seen in the luminal B BT-474 cells (ER+/PR+). **(b)** Relative *PADI2* mRNA levels in the cell lines compared to MCF10A. **(c)** Tumors from MMTV-neu mice (luminal subtype) show a ~15-fold increase in *PADI2* compared to normal mammary tissue. Both MMTV-*Wnt-1* hyperplastic mammary tissue and tumors (basal subtype) show elevated levels of *PADI2* compared to normal mammary tissue; however, *PADI2* expression levels in MMTV-*Wnt-1* tumors are ~10-fold less than those seen in the MMTV-neu tumors. *PADI2* mRNA levels were determined by qRT-PCR (TaqMan) using MCF10A cells as a reference and *GAPDH* normalization. Expression levels were analyzed using the 2 ^-ΔΔ C(t)^ method, and data are expressed as the mean ± SD from three independent experiments (* p < 0.05, ** p < 1 × 10^-6^). **(d)** RNA-seq analysis of 57 breast cancer cell lines shows that *PADI2* expression is significantly higher in luminal lines versus basal (* p = 0.007) and higher in basal versus basal-NM/claudin-low (** p = 0.002).

To test whether PADI2 displays a restricted expression pattern with respect to breast cancer subtype, we next investigated *PADI2* mRNA and protein expression in cell lines representing four common breast cancer subtypes: MCF7 (luminal A), BT-474 (luminal B), SK-BR-3 (HER2/ERBB2+), and MDA-MB-231 (basal). At the protein level, PADI2 was observed in both BT-474 (ER+, PR+, HER2/ERBB2+) and SK-BR-3 (ER-, PR-, and HER2/ERBB2 overexpressing) cell lines. Interestingly, the comparison of PADI2 and HER2/ERBB2 protein levels across these four cell lines supports the hypothesis that these two proteins are coexpressed (Figure 2a). While the PADI2 protein expression is not observed in MCF7 cells in Figure 2a, a longer exposure of this blot finds that PADI2 is weakly expressed in these cells (Additional file 1, Figure S1a). Analysis of *PADI2* transcript levels in these cell lines finds that, as expected, *PADI2* mRNA is sharply elevated in the BT-474 line (Figure 2b), and is ~2 fold higher that that seen in the MCF10DCIS cells (Additional file 1, Figure S1b) when compared to MCF10A cells. To test whether PADI2 expression is elevated in HER2/ERBB2 expressing cells *in vivo*, we next measured *PADI2* mRNA in normal murine mammary epithelium and in primary mammary tumors collected from MMTV-neu mice. Results indicate *PADI2* mRNA levels are ~15-fold higher in the HER2/ERBB2 overexpressing tumors compared to normal mammary tissue from littermate controls (Figure 2c). The ~15-fold increase in *PADI2* expression found in our study, compared to the ~4-fold increase found in the previous study [12], may simply reflect technical differences between the studies as we utilized TaqMan qRT-PCR compared to microarray analysis. We also investigated the level of *PADI2* mRNA in MMTV-*Wnt-1* mice, which is a basal mouse model of breast cancer [36-38]. The MMTV-*Wnt-1* model is unique in that it exhibits discrete steps in mammary tumorigenesis; the mammary glands are first hyperplastic, and then advance to invasive ductal carcinomas, finally culminating in fully malignant carcinomas that undergo metastasis [39]. Interestingly, we see that PADI2 levels are higher in the hyperplastic mammary glands (Figure 2c) when compared to normal mammary glands; however, the levels are less than those seen in the MMTV-neu tumors and are further reduced in the fully malignant MMTV-*Wnt-1* tumors.

**Figure 3:**
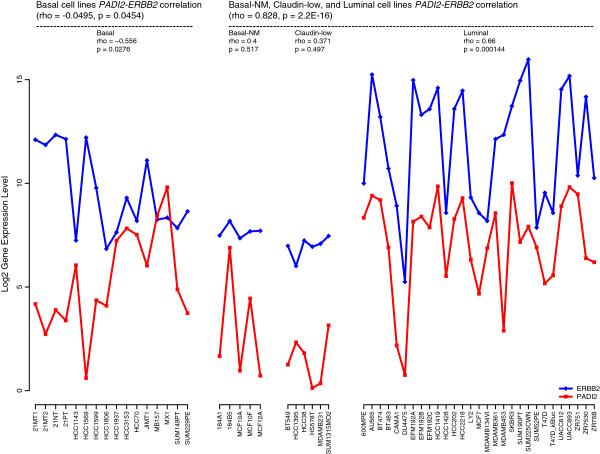
**RNA-seq analysis of *PADI2 *expression across 57 breast cancer cell lines shows subtype specific expression and high correlation with *HER2/ERBB2*.** The Spearman correlation between *PADI2* and *HER2/ERBB2* overexpression was highly significant across the luminal, basal-NM, and claudin-low cell lines (rho = 0.828, p = 2.2 × 10^-16^). A significant anti-correlation between *PADI2* and *HER2/ERBB2* was observed across the basal cell lines (rho = −0.495, p = 0.045).

To strengthen the hypothesis that PADI2 is primarily expressed in luminal breast cancer cell lines and is coexpressed with HER2/ERBB2, we next investigated *PADI2* mRNA levels by querying RNA-seq datasets collected from 57 breast cancer cell lines. A summary of *PADI2* expression in these lines is shown in the Additional file 2, Figure S2, with the most significant difference (p = 3.59 × 10^-5^) in *PADI2* expression across subtypes being found when luminal lines were compared with all non-luminal subtypes (Figure 2d). We then quantified the correlation between *PADI2* and *HER2/ERBB2* expression across the 57 cell lines. Results show that the correlation between *PADI2* and *HER2/ERBB2* overexpression is highly significant across the luminal, basal-NM (non-malignant), and claudin-low cell lines (rho = 0.828, p = 2.2 × 10^-16^) (Figure 3). Interestingly, a correlation between *PADI2* and *HER2/ERBB2* expression was not observed across the basal cell lines. In contrast, a significant anti-correlation was observed (rho = −0.495, p = 0.045), suggesting that the expression of these genes may be regulated by different mechanisms in these cell lines. Lastly, we queried the RNA-seq dataset to determine which genes were best correlated with *HER2/ERBB2* and *PADI2* expression in the luminal, basal-NM, and claudin-low lines to assess the relative strength of their coexpression. Only a single gene (*CCL17*) was as correlated with *PADI2* as *HER2/ERBB2* (rho = 0.832, p = 2.2 × 10^-16^), and *PADI2* represented the 13th most highly correlated gene with *HER2/ERBB2* (Table 1), thus suggesting co-regulation between HER2/ERBB2 and PADI2.

**Table 1:** **Top 13 genes correlating with *HER2/ERBB2 *expression**

**Symbol**	**Description**	**rho**
*PGAP3*	Post-GPI attachment to proteins 3 (PERLD1)	0.941
*CREB3L3*	CAMP responsive element binding protein 3-like 3	0.927
*C2orf54*	Chromosome 2 open reading frame 54	0.860
*EFCAB4A*	EF-hand calcium binding domain 4A	0.857
*ARHGAP8*	Rho GTPase activating protein 8	0.852
*GRB7*	Growth factor receptor-bound protein 7	0.851
*TMEM210*	Transmembrane protein 210	0.849
*CLDN4*	Claudin 4	0.847
*MB*	Myoglobin	0.846
*ELF3*	E74-like factor 3 (ets domain transcription factor, epithelial-specific )	0.842
*TRIM3*	Tripartite motif containing 3	0.839
*PRSS8*	Protease, serine, 8	0.829
*PADI2*	Peptidylarginine deiminase, type II	0.828

Genes identified by RNA-seq to be upregulated in HER2/ERBB2+ breast cancers were tested for correlation (Spearman’s rho) and the top 13 genes are ranked from top to bottom, with *PADI2* exhibiting the 13^th^ strongest correlation with *HER2/ERBB2* overexpression.

### Inhibition of PADI activity reduces cellular proliferation in breast cancer cell lines

**Figure 4:**
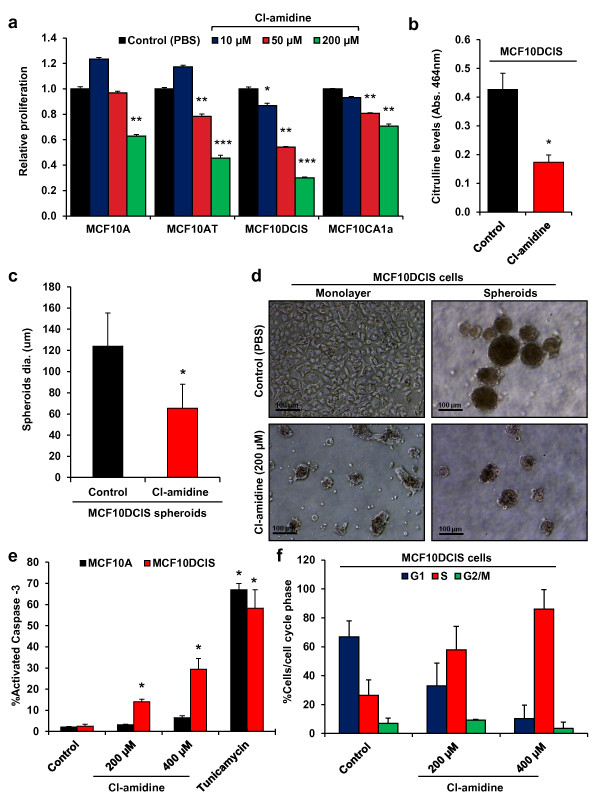
**PADI inhibitor Cl-amidine inhibits proliferation in breast cancer cell lines grown in monolayer and spheroid cultures. (a)** Relative mean fold difference in proliferation for the MCF10AT progression model cell lines at increasing concentrations of Cl-amidine after 5d treatment (n = 3, * p < 0.05, ** p < 0.005, *** p < 0.0005). **(b)** Citrulline levels for MCF10DCIS cells treated with 200 μM Cl-amidine were measured and compared to PBS control MCF10DCIS cells. Data represent (n = 3, * p < 0.005) **(c)** Multicellular spheroids were treated with 200 μM Cl-amidine and the diameter was measured and recorded in microns (n = 3, * p < 0.05). **(d)** Phase contrast images (10X) of MCF10DCIS cells grown in monolayer (2D) or multicellular spheroids (3D) treated with either vehicle (PBS) or 200 μM of Cl-amidine (scale bar = 100 μm). **(e** and **f)** MCF10A and MCF10DCIS cells were treated with different concentrations of Cl-amidine (0 μM, 200 μM, and 400 μM) and **(f)** 10μg/mL Tunicamycin, and analyzed by flow-cytometry. Data represents percent apoptotic cells (cleaved Caspase-3 positive) or percentage of cells in various phases of the cell cycle (DAPI), and are expressed as the mean ± SD from three independent experiments (* p < 0.005, ** p < 0.005).

To investigate whether PADI2 expression is important for breast cancer cell proliferation, we next tested whether the pharmacological inhibition of PADI2 activity negatively affects the growth of tumor cells *in vitro.* We utilized the small molecule inhibitor Cl-amidine for this study because we have previously shown that this drug binds irreversibly to the active site of PADIs, thereby blocking activity *in vitro* and *in vivo*[40]. Cl-amidine functions as a “pan-PADI” inhibitor as it blocks the activity of all active PADI family members (i.e. PADIs 1–4) with varying degrees of specificity [41]. Cultures from the MCF10AT cell line series were treated with 10 μM, 50 μM, or 200 μM of Cl-amidine, and the effects of the inhibitor on cell proliferation were quantified. Results show a dose-dependent decrease in the growth of all cell lines. Additionally, given that 200 μM Cl-amidine decreased the growth of MCF10DCIS cells by 75% (Figure 4a), this cell line appeared to be particularly affected by the inhibitor. Given the high level of PADI2 expression in the MCF10DCIS line, this finding suggests that PADI2 is likely playing an important role in the growth of MCF10DCIS cells. Importantly, while Cl-amidine also suppressed the growth of MCF10DCIS cells at lower concentrations, these doses did not inhibit the growth of the non-tumorigenic “normal” MCF10A line. These data suggest that Cl-amidine is not generally cytotoxic. In addition, citrulline levels in the Cl-amidine treated MCF10DCIS cells were significantly reduced, suggesting that the inhibitory effect of Cl-amidine was specifically due to the blockade of PADI activity (Figure 4b). In order to test the potential anti-tumor efficacy of Cl-amidine in a physiological model, we investigated the effects of this inhibitor on the growth of MCF10DCIS tumor spheroids. Spheroids grown from this cell line have been shown by others to form acinar-like structures that closely recapitulate the comedo-DCIS lesions that form in MCF10DCIS xenografts [18,20,42]. Results from our studies found that Cl-amidine treatment significantly reduces tumor spheroid diameter (Figures 4c). Representative images of the effects of Cl-amidine on the growth of MCF10DCIS monolayers and spheroids are shown in Figure 4d.

### Cl-amidine alters the expression of cell cycle associated genes and induces apoptosis

**Figure 5:**
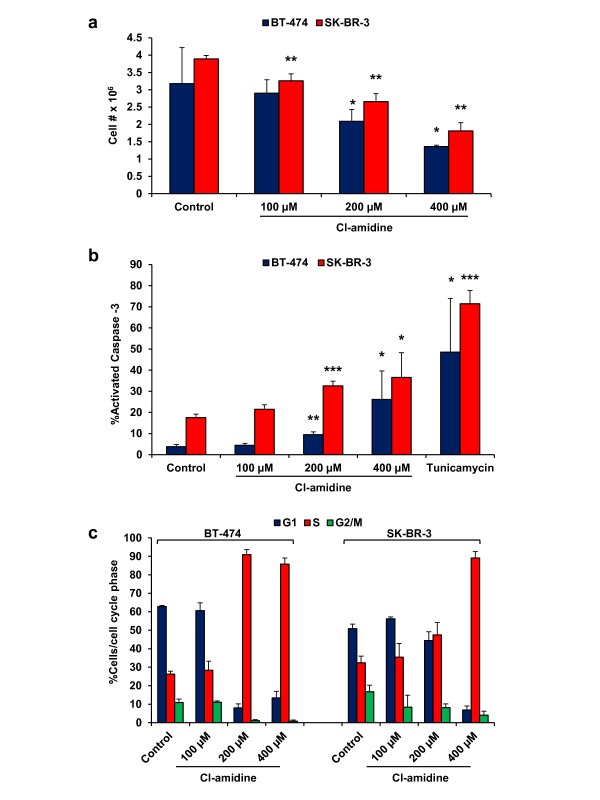
**HER2/ERBB2 expressing cell lines BT-474 and SK-BR-3 show decreased proliferation after treatment with Cl-amidine. (a)** BT-474 and SK-BR-3 cell lines were treated with increasing concentrations of Cl-amidine (0 μM, 100 μM, 200 μM, and 400 μM) over 4d and analyzed by flow-cytometry. Cell counts show a dose-dependent decrease in the growth of both BT-474 and SK-BR-3 (* p < 0.05, ** p < 0.001). **(b)** Apoptosis levels (cleaved Caspase-3 positive) significantly increase over the control in both BT-474 and SK-BR-3 cells after 200 μM and 400 μM of Cl-amidine. Tunicamycin (10μg/mL ) is shown as a control for apoptosis (* p < 0.05, ** p < 0.01, *** p < 0.001). **(c)** Cell cycle analysis of Cl-amidine treated cells (DAPI) indicates S-phase arrest occurs in both BT-474 and SK-BR-3 cell lines. Data are expressed as the mean ± SD from three independent experiments.

The observed effects of Cl-amidine on cell proliferation suggested that this drug might affect tumor growth by altering the expression of genes involved in cell cycle progression. To test this hypothesis, mRNA from the Cl-amidine treated and control MCF10DCIS cells was examined for the expression of cell cycle associated genes using the RT^2^ Profiler PCR Cell Cycle Array via qRT-PCR. Using a threshold value of 2-fold expression change and a statistical significance of p < 0.05, we found that Cl-amidine affected the expression of a subset of genes (for the full unsorted list see Additional file 3, Table S1), with the top 10-upregulated and -downregulated genes presented in Table 2. Importantly, previous studies have shown that increased expression of *GADD45α*, the second most highly upregulated gene in our study, leads to cell cycle arrest and apoptosis in a range of cell types, including breast cancer cells [43]. This observation suggested that, in addition to affecting cell cycle gene expression (e.g. *p21*), Cl-amidine might also alter MCF10DCIS cell growth by inducing apoptosis. To test this hypothesis, we next treated MCF10A and MCF10DCIS cells with increasing concentrations of Cl-amidine for 4 days. Cells were fixed and labeled with anti-activated Caspase-3 antibody or DAPI, and then analyzed by flow-cytometry. Results show that Cl-amidine treatment significantly increased the percent of apoptotic MCF10DCIS cells in a dose-dependent manner (Figure 4e). In contrast, the MCF10A cells were largely unaffected. Furthermore, we also show that treatment of MCF10DCIS cells with Cl-amidine appears to induce cell cycle arrest in S-phase (Figure 4f). Lastly, we wanted to see whether the increase in apoptosis occurs earlier after treatment, so we tested the cells again following 2 days of treatment, but were unable to see any effect (Additional file 4, Figure S3a). However, this was not surprising, as the effects of Cl-amidine are most pronounced after 3 days of treatment (data not shown). Taken together, it appears that Cl-amidine treatment after 4 days leads to S-phase coupled apoptosis, which is an intrinsic mechanism that prevents DNA replication of a damaged genome in a mammalian cell [44]. We also tested the effects of Cl-amidine on HER2/ERBB2 overexpressing cell lines BT-474 and SK-BR-3. Again, we see a reduction in cell growth (Figure 5a) and an increase in apoptosis (Figure 5b) that is coupled to S-phase cell cycle arrest (Figure 5c) for both BT-474 and SK-BR-3. These results show that Cl-amidine is effective in inhibiting the growth of luminal-HER2/ERBB2+ cell lines, BT-474 and SK-BR-3, and agree with previously reported data on Cl-amidine inhibition of growth in MCF7 cells [8,9,11,45]. We wanted to test whether there would be any effect on a basal cell line, and chose MDA-MB-231 for comparison. Surprisingly, we see an effect on both cell growth and apoptosis (Additional file 4, Figure S3b and c), albeit a smaller effect on apoptosis than we see in BT-474 and SK-BR-3. While this is interesting, and perhaps suggests the expression of a different PADI family member in this basal cell line, we have focused on PADI2 expressing cancers for this study, which are predominantly luminal and HER2/ERBB2 expressing. Taken together, these results suggest that Cl-amidine blocks the growth of MCF10DCIS cells by inducing cell cycle arrest and apoptosis. This prediction is supported by our previous finding that Cl-amidine can also drive apoptosis in lymphocytic cell lines *in vitro*[3]. Importantly, the lack of an apoptotic effect in MCF10A cells suggests that Cl-amidine may primarily target tumor cells for killing. Consistent with this possibility is the fact that Cl-amidine did not affect the growth of non-tumorigenic NIH3T3 cells and HL60 granulocytes [11].

**Table 2:** Top 10 cell cycle genes up- and down-regulated in MCF10DCIS cells after Cl-amidine treatment

**Symbol**	**Description**	**Fold change**	**p-value**
*Genes upregulated*			
*CDKN1A*	Cyclin-dependent kinase inhibitor 1A (p21, Cip1)	17.68	1.06E-03
*GADD45A*	Growth arrest and DNA-damage-inducible, alpha	13.53	3.57E-03
*ATM*	Ataxia telangiectasia mutated	9.61	6.91E-04
*DIRAS3*	DIRAS family, GTP-binding RAS-like 3	8.87	2.41E-04
*HERC5*	Hect domain and RLD 5	8.20	2.31E-03
*RAD17*	RAD17 homolog (S. pombe)	7.50	2.20E-05
*CUL2*	Cullin 2	6.57	7.37E-04
*CCNE1*	Cyclin E1	6.18	5.51E-04
*ATR*	Ataxia telangiectasia and Rad3 related	5.83	1.77E-04
*CCNT2*	Cyclin T2	5.68	1.15E-04
*Genes downregulated*			
*MCM5*	Minichromosome maintenance complex component 5	−8.45	3.23E-02
*CDK1*	Cyclin-dependent kinase 1	−8.14	2.88E-03
*CDC20*	Cell division cycle 20 homolog (S. cerevisiae)	−7.90	2.00E-06
*GTSE1*	G-2 and S-phase expressed 1	−6.62	4.84E-03
*MCM2*	Minichromosome maintenance complex component 2	−6.40	7.55E-04
*MKI67*	Antigen identified by monoclonal antibody Ki-67	−5.11	7.60E-05
*CCNF*	Cyclin F	−5.07	1.10E-02
*BIRC5*	Baculoviral IAP repeat containing 5 (Survivin)	−4.49	1.10E-02
*BCL2*	B-cell CLL/lymphoma 2	−4.14	1.70E-05
*CCND2*	Cyclin D2	−3.74	3.95E-02

MCF10DCIS cells were treated with 200 μM Cl-amidine after seeding (5 × 10^4^), and total RNA was collected 5d post-seeding. The mRNA for each group was tested on the RT^2^ Profiler PCR Cell Cycle Array (SABiosciences Corporation, catalog number PAHS-020A). The PCR array was designed to study the profile of 84 human cell cycle related genes. Using a threshold value of 2-fold expression change and a statistical significance of p < 0.05, we found that Cl-amidine affected the expression of a subset of genes, with the top 10 up- and down-regulated genes displayed here.

### PADI2 is highly expressed in the luminal epithelium of xenograft tumors derived from MCF10DCIS cells

**Figure 6:**
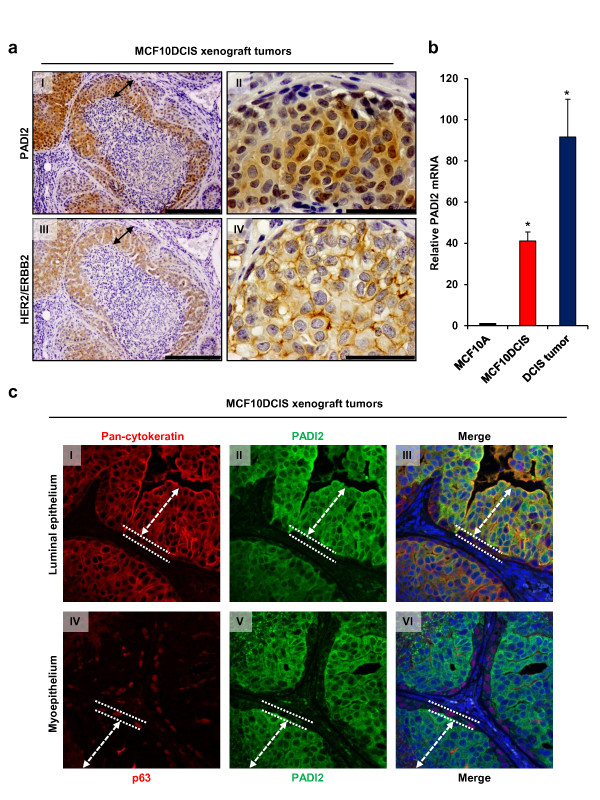
**PADI2 is expressed in MCF10DCIS xenograft tumors and localizes to the luminal epithelium. (a)** MCF10DCIS cells (1 × 10^6^) were injected subcutaneously into female nude (nu/nu) mice (Charles River) and comedo-DCIS tumors formed after 2 weeks. Tumor sections were probed with an (I, II) anti-PADI2 (1:100) or (III, IV) anti-ERBB2 antibody (1:100), and counterstained with hematoxylin. The black arrows indicate the luminal epithelium of the duct-like structures in DCIS tumors (I, III – 20X, scale bar = 200 μm; II, IV – 100X, scale bar = 50 μm). While the HER2/ERBB2 staining is predominantly cytoplasmic (IV), there are some nuclei staining positively for PADI2 (II). **(b)** Tumors were collected; two sections from each mouse were sampled and total RNA was isolated. *PADI2* mRNA levels were determined by qRT-PCR (TaqMan) using MCF10A cells as a reference and *GAPDH* normalization. *PADI2* levels were higher in the tumor samples than normally found in the MCF10DCIS cells. Data were analyzed using the 2 ^-ΔΔ C(t)^ method, and are expressed as the mean ± SD from three independent experiments (* p < 0.005). **(c)** Immunofluorescence staining (40X) shows that PADI2 is expressed in the luminal but not myoepithelium cells in MCF10DCIS tumors. Tumors were probed with anti-PADI2 (II, III, V, VI – green fluorescent signal), anti-cytokeratin (I, III – luminal marker – red fluorescent signal), and anti-p63 (IV, VI – myoepithelial marker – red fluorescent signal). Nuclei were stained with DAPI (blue fluorescent signal). The dashed arrows delineate the luminal epithelium layer, while the dashed straight line delineates the myoepithelium layer, which is adjacent to the basement membrane. In the merged images, the co-localization of cytokeratin and PADI2 can be seen in the luminal epithelium (III), while in contrast, PADI2 is absent from the p63 stained myoepithelium (VI).

Given that PADI2 expression is elevated in the MCF10DCIS cell line, we investigated PADI2 expression and localization in primary tumors derived from MCF10DCIS-injected mouse xenografts. Previous studies have shown that when MCF10DCIS cells are injected into the mammary fat-pad of immunodeficient nude (nu/nu) mice, tumors develop within 2–3 weeks. These tumors faithfully recapitulate the human comedo-DCIS condition, with the basement membrane limiting duct-like structure being comprised of an outer myoepithelial layer, an inner layer of luminal epithelial cells, and a central necrotic lumen [18,19,46,47]. We chose to use subcutaneous injections instead of orthotopic or intraductal [48] methods, as previous work by Hu et al. showed that the progression and phenotype of the MCF10DCIS tumors grown subcutaneously in the mammary fat pad were highly similar to human high-grade comedo-DCIS tumors [19]. In our study, we found that PADI2 protein expression was restricted to the luminal epithelium of the duct-like structures in the MCF10DCIS xenografts, and was not observed in the stromal tissue or the necrotic core (Figure 6a, panel I and II). At the subcellular level, PADI2 appears to be expressed in both the cytoplasmic and nuclear compartments of luminal epithelial cells (Figure 6a, panel II). This observation supports our recent findings that PADI2 can be targeted to the nucleus of both human normal mammary tissue and breast cancer cells [49] and regulate gene activity via citrullination [49,50].

Next, we examined whether the observed correlation between PADI2 and HER2/ERBB2 expression also occurred *in vivo.* We found that both HER2/ERBB2 and PADI2 were expressed within the luminal epithelium of MCF10DCIS tumors (Figure 6a, panel III and IV). Interestingly, a previous report by Behbod et. al. found low levels of HER2/ERBB2 in MCF10DCIS tumors that were grown intraductally. The disparity between this data and our data may be due to differences in the microenvironment. We then quantified *PADI2* mRNA in the MCF10DCIS xenografts by qRT-PCR, and found that *PADI2* levels were significantly higher in the tumors when compared to monolayer cultures (Figure 6b). We also carried out immunofluorescence (IF) analysis of these tumors to examine PADI2 intratumoral localization, and found that PADI2 protein expression appears entirely limited to cytokeratin-positive luminal epithelial cells (Figure 6c, panel I and III, and Additional file 5, Figure S4), while no detectable PADI2 signal was observed in the p63 positive myoepithelial cells (Figure 6c, panel IV and VI, and Additional file 5, Figure S4).

### Treatment of MCF10DCIS xenografts with Cl-amidine suppresses tumor growth

**Figure 7:**
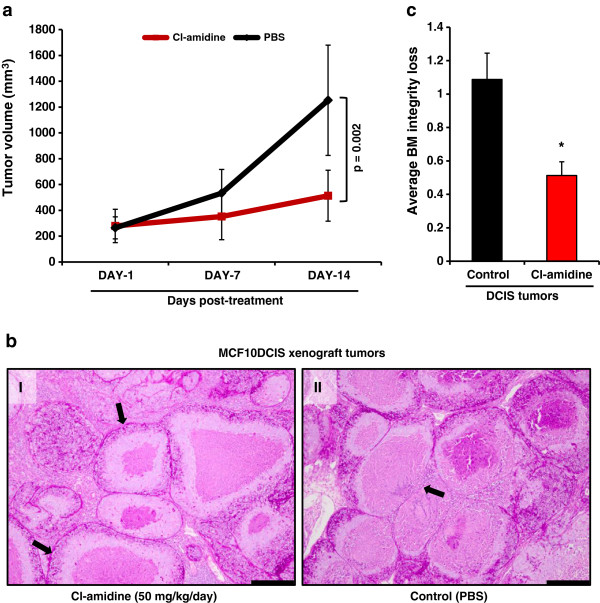
**Cl-amidine decreases the growth of MCF10DCIS tumors in a xenograft model of comedo-DCIS. (a)** Average tumor volumes after 1, 7, and 14 days of daily IP injections of Cl-amidine (50mg/kg/day) or PBS as vehicle control. MCF10DCIS cells (1 × 10^6^) were injected subcutaneously into female nude (nu/nu) mice (Charles River). After 2 weeks of tumor growth (tumor volumes ~100-200 mm^3^), mice were randomly treated with intraperitoneal injections of Cl-amidine at 50 mg/kg/day (n = 7) or PBS as a vehicle control (n = 7). Tumor volume was measured weekly by digital caliper, and the differences between tumor volumes were evaluated by the non-parametric Mann–Whitney–Wilcoxon test (MWW). Results are reported as mean ± SD (Day 14, p = 0.002). **(b)** PAS stained sections (10X, scale bar = 200 μm) from representative treated (I) and control tumors (II), arrows in (I) show an intact basement membrane (BM), while the arrow in (II) shows breaching of the BM with infiltrating leukocytes. **(c)** BM integrity was scored on a scale from 1–3 by S.M., with values being expressed as the mean ± SD. The treated tumors have a lower score, indicating a higher level of BM integrity (* p < 0.05 MWW).

Given the inhibitory effects of Cl-amidine on MCF10-DCIS monolayer and spheroid growth, we next tested whether the treatment of mice with this inhibitor would suppress the growth of MCF10DCIS-derived tumors. For this study, mouse fat-pads were injected with MCF10DCIS cells (1 × 10^6^) and the tumors were allowed to establish and grow for ~2 weeks as described previously [18-20,46]. Mice were randomly assigned into treatment or control groups and administered daily intra-peritoneal (IP) injections of either Cl-amidine (50 mg/kg/day) or vehicle (PBS). Note, that the choice of dose and route of administration were based on the previous demonstration that Cl-amidine reduces disease severity in the murine collagen induced arthritis model of rheumatoid arthritis [5]. Treatment continued for 14 days, at which point the tumors were harvested. Results from our xenograft study show that Cl-amidine treatment (n = 7/group) caused a significant reduction in the size of the tumors (Figures 7a). Moreover, the analysis of tumor morphology by H&E and PAS staining shows that, while tumors from the sham-injected group displayed an advanced, potentially invasive, tumor phenotype (Figure 7b, panel II), tumors from the Cl-amidine treated group (Figure 7b, panel I) were much more benign in appearance. Furthermore, the basement membrane of Cl-amidine treated tumors remained largely intact (Figure 7c) and had considerably less membrane breaching and leukocyte infiltration compared to the control group. These findings suggest that PADI2 plays an important role in comedo-DCIS progression and that the inhibition of PADI activity can suppress tumor progression *in vivo*. 

## Discussion

In this study, we show that PADI2 is specifically upregulated during mammary tumor progression and that the PADI inhibitor, Cl-amidine, is effective in inhibiting the growth of PADI2 overexpressing cell lines in both 2D and 3D cultures. In addition, we demonstrate here for the first time that Cl-amidine is successful in suppressing tumor growth in a xenograft mouse model of comedo-DCIS. Lastly, we document that PADI2 expression is highly correlated with HER2/ERBB2 overexpressing and luminal subtype breast cancers.

Given the previous correlations between PADI2 and the HER2/ERBB2 oncogene, the goal of this study was to carry out an initial test of the hypothesis that PADI2 plays a role in breast cancer progression. To accomplish this, we utilized the well-established MCF10AT model [16,17] and found that PADI2 expression was highly upregulated in MCF10DCIS cells, a cell line that forms comedo-DCIS lesions that spontaneously progress to invasive tumors [30,46]. Our finding that PADI2 expression is highest in comedo-DCIS lesions (defined by their necrotic centers) was perhaps not too surprising, given the close association of PADIs with inflammatory events. We are currently investigating the potential links between inflammatory signaling in these MCF10DCIS lesions and PADI2 activity.

Interestingly, PADI2 expression in the MCF10AT series coincided with HER2/ERBB2 upregulation which, again, was not entirely unexpected given previous reports correlating *PADI2* expression with *HER2/ERBB2*[15]. While we did find that HER2/ERBB2 and PADI2 protein expression correlated well across the MCF10AT cell lines, PADI2 protein levels are particularly high in the MCF10DCIS line, relative to HER2/ERBB2. We cannot currently explain this finding; however, it is possible that cell-line-specific factors are stabilizing the PADI2 transcript, thus allowing for increased protein expression [51,52].

While our data show a potential relationship between PADI2 and HER2/ERBB2 in the MCF10AT model, we wanted to examine this correlation at higher resolution. To accomplish this we queried our RNA-seq dataset of 57 breast cancer cell lines with known subtype and HER2/ERBB2 status and found that: (a) *PADI2* expression is highest in luminal cell lines and that (b) *PADI2* expression is highly correlated with HER2/ERBB2 overexpression across the basal-NM, claudin-low, and luminal lines. The observation that *PADI2* is upregulated in the luminal subtype confirms previous gene expression data where *PADI2* was identified as one of the top upregulated genes in luminal breast cancer lines compared to basal lines [13,14]. In order to test whether the observed correlation between *PADI2* and *HER2/ERBB2* would be retained at the protein level, we also tested a small sample of cell lines representing the four common breast cancer subtypes and found that PADI2 expression was only observed in the HER2/ERBB2+ BT-474 and SK-BR-3 lines. However, we did observe some discordance seen between *PADI2* transcript and protein levels, but we predict this difference may be due to post-transcriptional regulatory mechanisms. This prediction is based, in part, upon the observation that PADI2 possesses a long 3’UTR [53] that contains several AU-rich elements [54,55] that have been shown to bind the stabilizing regulatory factor HuR [56]. HuR binding has been shown to enhance the stability of mRNAs involved in proliferation [57-59], while also playing a role in breast cancer, as cytoplasmic accumulation of HuR promotes tamoxifen resistance in BT-474 cells [60] and the stability of *HER2/ERBB2* transcripts in SK-BR-3 cells [52]. Interestingly, from these studies, the level of HuR was reported to be high in both BT-474 and SK-BR-3 cells, while it was relatively low in MCF7 cells. It is important to note that while we observed low levels of PADI2 protein expression in MCF7 (Additional file 1: Figure S1a), recent work from our lab has confirmed the expression of PADI2 in MCF7 cells [49,50].

We also examined two mouse models of mammary tumorigenesis, the luminal MMTV-neu and the basal MMTV-*Wnt-1*, and found that, as predicted, PADI2 levels are highest in the HER2/ERBB2 overexpressing MMTV-neu mice compared to normal mammary tissue and to hyperplastic and primary MMTV-*Wnt-1* tumors. Taken together, these findings indicate that PADI2 is predominantly expressed in luminal epithelial cells, and that there appears to be a strong relationship between PADI2 and HER2/ERBB2 expression in breast cancer. Subsequent studies are now underway to test whether PADI2 plays a functional role in HER2/ERBB2 driven breast cancers, potentially by functioning as an inflammatory mediator.

Previous studies have shown that the inhibition of PADI enzymatic activity by Cl-amidine is effective in decreasing the growth of several cancer cell lines (i.e. HL-60, HT-29, U2OS, and MCF7 cells), and that administering the drug in combination with doxorubicin or the HDAC inhibitor SAHA can have synergistic cytotoxic effects on cells [8,9,11,45]. Cl-amidine is highly specific for all PADI enzymes, with dose-dependent cytotoxicity and little to no effect in non-cancerous cell lines (i.e. HL-60 granulocytes and NIH3T3 cells) [11]. Our studies expand on these previous results by showing that Cl-amidine suppresses the growth of the transformed lines of the MCF10AT model, especially the MCF10DCIS cell line, in both 2D and 3D cultures. In addition, we show for the first time that Cl-amidine is successful in treating tumors *in vivo* using a mouse model of comedo-DCIS from xenografted MCF10DCIS cells. Given that the loss of basement membrane integrity is an important event during the progression of DCIS to invasive disease, it is significant that Cl-amidine treated xenografts maintain their basement membrane integrity and show reduced leukocytic infiltration across the basement membrane compared to the control group. These observations suggest that Cl-amidine treatment might enhance the ability of tumor ductular myoepithelial cells to deposit continuous and organized basement membranes.

While we chose the subcutaneous model of MCF10DCIS tumorigenesis, future studies on the effect of Cl-amidine could examine alternate methods of transplantation, such as the previously described intraductal method [48]. In addition, different models of DCIS could be examined, such as xenografted SUM-225 cells, which show high *HER2/ERBB2* and *PADI2* levels (see Figure 3 for relative levels). Of note, we found that while Cl-amidine suppressed tumor growth, the drug was well tolerated by mice in this study. Similarly, our previous work found that doses of Cl-amidine up to 75 mg/kg/day in a mouse model of Colitis [3], and up to 100 mg/kg/day in a mouse model of RA [5], were well-tolerated without side effects. Further work into studying the pharmacokinetics and biodistribution of Cl-amidine, or perhaps the development of an isozyme specific inhibitor of PADI2, will be an important step in helping to find a potent drug for the treatment of DCIS patients.

The actual mechanisms by which Cl-amidine reduces cellular proliferation have yet to be fully elucidated, though evidence here suggests that PADI2 may play a role (direct or indirect) in regulating the expression of both cell cycle and tumor promoting genes. Previous reports have shown that Cl-amidine effectively upregulates a number of p53-regulated genes, including *p21*, *PUMA*, and *GADD45*[8,45]. Our qRT-PCR cell cycle array results confirm that two of these genes, *p21* and *GADD45α*, are upregulated after treatment of MCF10DCIS cells with Cl-amidine by 17.68- and 13.53-fold, respectively. Furthermore, we have identified additional genes downregulated by Cl-amidine, including *MKI67*, *MCM5*, and *MCM2*, each with known functions in cancer progression**.** We have also quantitatively analyzed for apoptosis levels (Caspase-3) after Cl-amidine treatment via flow-cytometry, and see a dose-dependent decrease in proliferation and increase in apoptosis. Moreover, we also show that the cells arrest in S-phase after Cl-amidine treatment, thus leading to S-phase coupled apoptosis, which is a known response to DNA damage [44]. Taken together, the observed inhibitory effects of Cl-amidine on tumor growth may be due to the suppression of genes involved in oncogenesis and the activation of genes involved in apoptosis, though additional work is needed to define the mechanisms behind these potential relationships.

## Conclusions

In summary, we provide here an important new line of evidence demonstrating that PADI2 may play a role in the oncogenic progression of cancer and, in particular, breast cancer. Using the MCF10AT model, we show that PADI2 is highly upregulated following transformation at both the mRNA and protein level, with highest levels in the cell line that recapitulates human comedo-DCIS. Furthermore, we show that, across a wide array of breast cancer cell lines, *PADI2* is specifically overexpressed in the luminal subtype, while also being highly correlated with HER2/ERBB2 overexpression. This observation suggests that PADI2 may function as a biomarker for HER2/ERBB2+ lesions. Lastly, our preclinical mouse xenograft study suggests that the PADI inhibitor, Cl-amidine, could potentially be utilized as a therapeutic agent for the treatment of comedo-DCIS tumors.

## Abbreviations

PADI: Peptidylarginine deiminase; EGF: Epidermal growth factor; HER2: Human epidermal growth factor receptor 2; ERBB2: v-erb-b2 avian erythroblastic leukemia viral oncogene homolog 2; ER: Estrogen receptor; PR: Progesterone receptor; MMTV: Mouse mammary tumor virus; DCIS: Ductal carcinoma *in situ*; RNA-seq: Ribonucleic acid sequencing; ARE: AU-rich element; ALEXA-seq: Alternative expression analysis by sequencing; RA: Rheumatoid arthritis; COPD: Chronic obstructive pulmonary disease; qRT-PCR: Quantitative real-time polymerase chain reaction; IHC: Immunohistochemistry; IF: Immunofluorescence; H&E: Hematoxylin and eosin; PAS: Periodic acid-Schiff; BAEE: α-N-benzoyl-L-arginine ethyl ester.

## Competing interests

The authors declare that they have no competing interests.

## Authors’ contributions

JLM, SM, OLG, HCB, BDC, AMP, and LJA all acquired primary data and helped in the analysis of the research found in this manuscript. JLM was involved in the design of the study and drafted the manuscript, in addition to performing molecular genetic studies on both *in vitro* and *in vivo* models. SM generated the 3D-spheroids and performed pathological analyses. OLG performed RNA-seq and ALEXA-seq, and statistical analysis on the data from the collection of breast cancer cell lines. HCB participated in generating MCF10DCIS xenografts and in the *in vivo* drug study. BDC performed IF experiments and helped with data analysis. AMP assayed PADI activity/citrulline levels. LJA performed flow-cytometry experiments and FACS analysis. VS and CPC designed and synthesized the Cl-amidine used for the experiments. LRH provided MMTV mouse models and helped with data analysis. SM, OLG, BDC, and PRT helped revise the manuscript. SAG helped in the design of the study and contributed to the manuscript revision. PRT, JWG, and SAG supervised the study. All authors read and approved the final manuscript.

## Pre-publication history

The pre-publication history for this paper can be accessed here:

http://www.biomedcentral.com/1471-2407/12/500/prepub

## Supplementary Material

Additional file 1 **Figure S1.** Comparative expression levels of MCF10DCIS and HER2/ERBB2 expressing BT-474 and SK-BR-3 cell lines. (A) Overexposure of image in Figure 2a, showing that there are low levels of PADI protein found in the MCF7 cell line. (B) MCF10DCIS *PADI2* levels are about half that of BT-474 cells, with SK-BR-3 *PADI2* levels being about half that of MCF10DCIS cells. These *PADI2* levels recapitulate the relationship seen at the protein level. Total RNA was isolated from MCF10A, MCF10DCIS, BT-474, and SK-BR-3 cell lines and *PADI2* mRNA levels were determined by qRT-PCR (TaqMan) using MCF10A cells as a reference and *GAPDH* normalization. Data were analyzed using the 2 ^-ΔΔ C(t)^ method and are expressed as the mean ± SD from three independent experiments (* p < 0.005). Click here for file

Additional file 2 **Figure S2.***PADI2* gene-level expression compared to distribution of all genes across 57 breast cancer cell lines. *PADI2* mRNA expression levels across 57 breast cancer cell lines were measured by RNA-seq. *PADI2* levels are shown relative to all other genes in each cell line. *PADI2* is most highly expressed in the luminal lines (26/29 above background). PADI2 levels are significantly different in luminal cell lines when compared to all non-luminal cell lines (p = 3.59 × 10^-5^). Click here for file

Additional file 3 **Table S1.** Complete list of genes from RT2 Profiler PCR Cell Cycle Array with fold change values for MCF10DCIS cells after Cl-amidine treatment. MCF10DCIS cells were treated with 200 μM Cl-amidine and RNA collected 5d post-seeding. The RT^2^ Profiler PCR Cell Cycle Array (SABiosciences Corporation, catalog number PAHS-020A) profiles the gene expression of 84 human cell cycle-related genes. Click here for file

Additional file 4 **Figure S3.** Flow-cytometry analysis of apoptosis in MCF10A and MCF10DCIS cell lines, and both proliferation/cell-growth and apoptosis in MDA-MB-231 cells. (A) MCF10A and MCF10DCIS cells were treated with different concentrations of Cl-amidine (0 μM, 200 μM, and 400 μM) and 10μg/mL Tunicamycin, and analyzed by flow-cytometry. Data represents percent apoptotic cells (cleaved Caspase-3 positive) after 2d and are expressed as the mean ± SD from three independent experiments (* p < 0.05, ** p < 0.001). (B) MDA-MB-231 cells were treated with increasing concentrations of Cl-amidine (0 μM, 100 μM, 200 μM, and 400 μM) over 4d and analyzed by flow-cytometry. Cell counts (DAPI) show a dose-dependent decrease in the growth (* p < 0.001). (C) Apoptosis levels (cleaved Caspase-3 positive) significantly increase over the control only after treatment with 400 μM of Cl-amidine. Tunicamycin (10μg/mL ) is shown as a control for apoptosis (* p < 0.01). Data are expressed as the mean ± SD from three independent experiments. Click here for file

Additional file 5 **Figure S4.** Immunofluorescence staining of MCF10DCIS xenografts for PADI2, luminal epithelium (pan-cytokeratin), and myoepithelium (p63). (A) MCF10DCIS cells (1 × 10^6^) were injected subcutaneously into female nude (nu/nu) mice (Charles River) and comedo-DCIS tumors formed after 2 weeks. Tumors were probed with anti-PADI2 (green fluorescent signal), anti-cytokeratin (luminal marker – red fluorescent signal), and anti-p63 (myoepithelial marker – red fluorescent signal). Nuclei were stained with DAPI (blue fluorescent signal). Immunofluorescence staining (63X) shows that PADI2 is expressed in the luminal but not myoepithelium cells in MCF10DCIS tumors. In addition, there is some evidence for PADI2 expression in the nucleus. Click here for file
