# Supplementary figures and images for: Identification of PADI2 as a potential breast cancer biomarker and therapeutic target
Source: BMC Cancer. 2012 Oct 30;12:500. doi: 10.1186/1471-2407-12-500 (PMC3571905; doi:10.1186/1471-2407-12-500)

## Slide 1
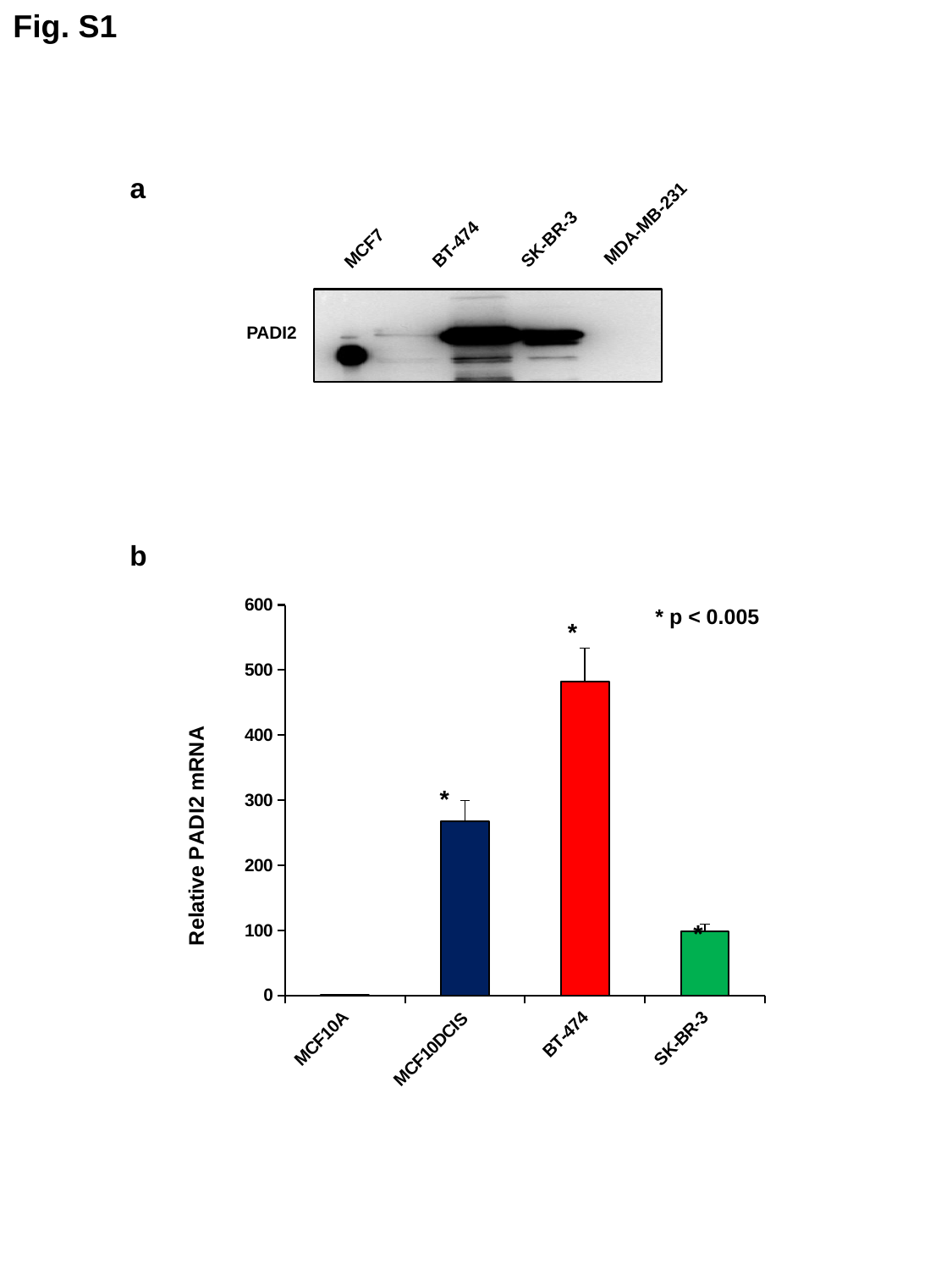

Fig. S1
a
MDA-MB-231
SK-BR-3
BT-474
MCF7
PADI2
b
### Chart
| Category | PADI2 |
|---|---|
| MCF10A | 1.0000000000000002 |
| MCF10DCIS | 268.2032408678935 |
| BT-474 | 482.52879245628384 |
| SK-BR-3 | 98.16771949098236 |* p < 0.005

Supplement: Additional file 1 — Figure S1. Comparative expression levels of MCF10DCIS and HER2/ERBB2 expressing BT-474 and SK-BR-3 cell lines. (A) Overexposure of image in Figure 2a, showing that there are low levels of PADI protein found in the MCF7 cell line. (B) MCF10DCIS PADI2 levels are about half that of BT-474 cells, with SK-BR-3 PADI2 levels being about half that of MCF10DCIS cells. These PADI2 levels recapitulate the relationship seen at the protein level. Total RNA was isolated from MCF10A, MCF10DCIS, BT-474, and SK-BR-3 cell lines and PADI2 mRNA levels were determined by qRT-PCR (TaqMan) using MCF10A cells as a reference and GAPDH normalization. Data were analyzed using the 2 -ΔΔ C(t) method and are expressed as the mean ± SD from three independent experiments (* p < 0.005). [file 1471-2407-12-500-S1.pptx]

## Slide 1
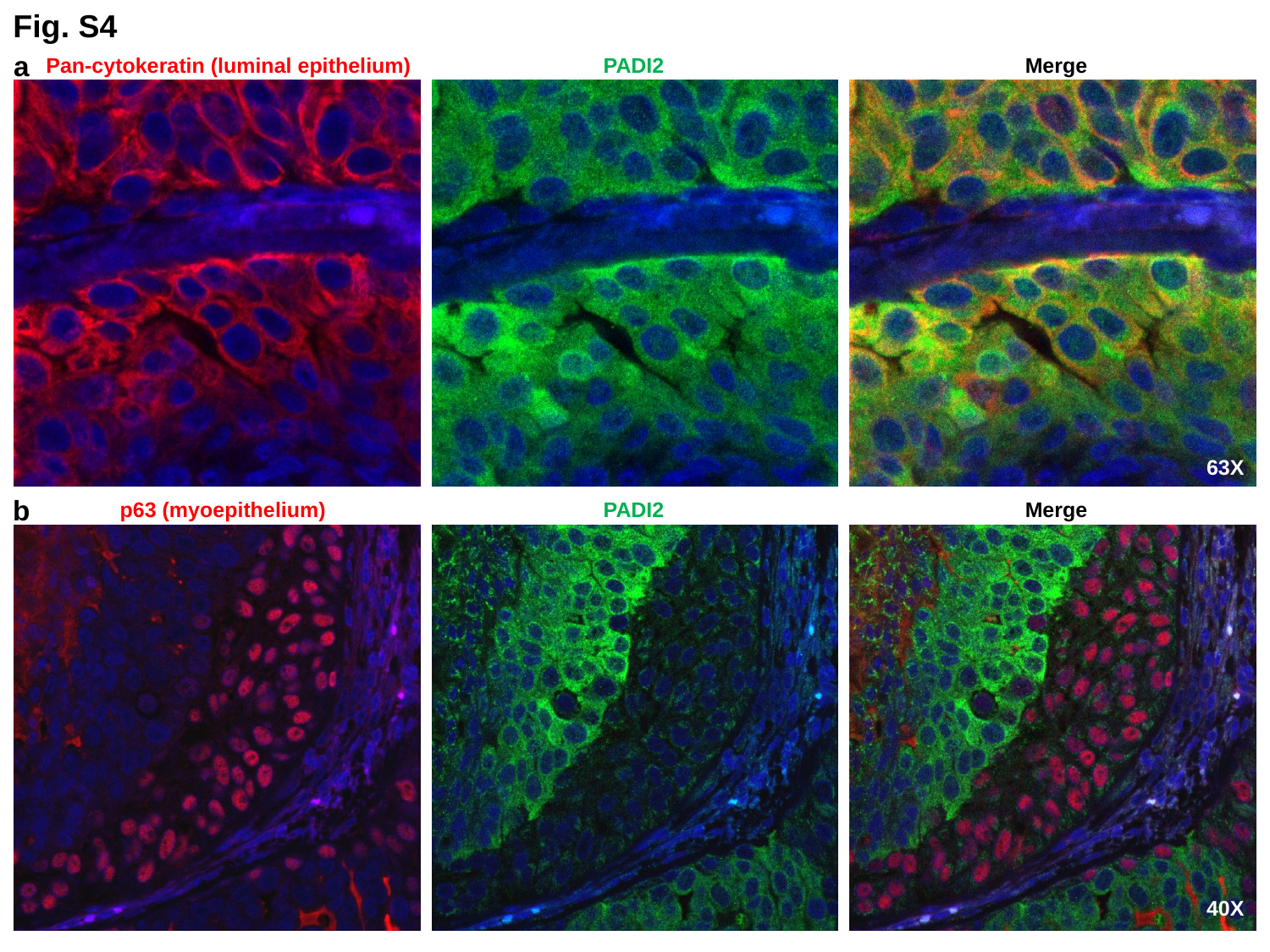

Fig. S4
a
Pan-cytokeratin (luminal epithelium)
PADI2
Merge
63X
b
p63 (myoepithelium)
PADI2
Merge
40X

Supplement: Additional file 5 — Figure S4. Immunofluorescence staining of MCF10DCIS xenografts for PADI2, luminal epithelium (pan-cytokeratin), and myoepithelium (p63). (A) MCF10DCIS cells (1 × 10^6^) were injected subcutaneously into female nude (nu/nu) mice (Charles River) and comedo-DCIS tumors formed after 2 weeks. Tumors were probed with anti-PADI2 (green fluorescent signal), anti-cytokeratin (luminal marker – red fluorescent signal), and anti-p63 (myoepithelial marker – red fluorescent signal). Nuclei were stained with DAPI (blue fluorescent signal). Immunofluorescence staining (63X) shows that PADI2 is expressed in the luminal but not myoepithelium cells in MCF10DCIS tumors. In addition, there is some evidence for PADI2 expression in the nucleus. [file 1471-2407-12-500-S5.pptx]
