# Supplementary material for: Identification of PADI2 as a potential breast cancer biomarker and therapeutic target
Source: BMC Cancer. 2012 Oct 30;12:500. doi: 10.1186/1471-2407-12-500 (PMC3571905; doi:10.1186/1471-2407-12-500)
Supplement: Additional file 2 — Figure S2.PADI2 gene-level expression compared to distribution of all genes across 57 breast cancer cell lines. PADI2 mRNA expression levels across 57 breast cancer cell lines were measured by RNA-seq. PADI2 levels are shown relative to all other genes in each cell line. PADI2 is most highly expressed in the luminal lines (26/29 above background). PADI2 levels are significantly different in luminal cell lines when compared to all non-luminal cell lines (p = 3.59 × 10^-5^). [file 1471-2407-12-500-S2.pdf]

Fig. S2

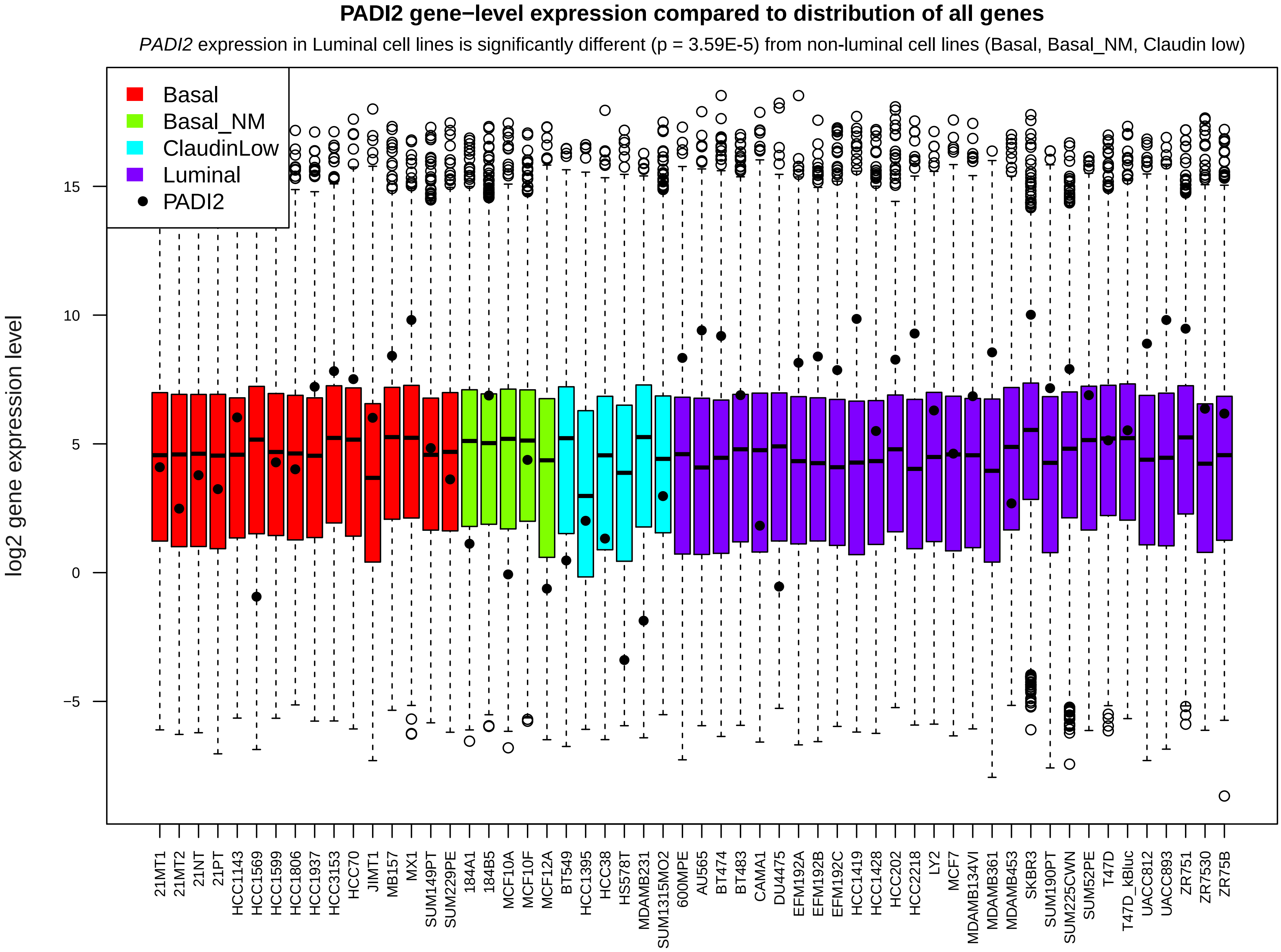
PADI2 gene−level expression compared to distribution of all genes
PADI2 expression in Luminal cell lines is significantly different (p = 3.59E-5) from non-luminal cell lines (Basal, Basal_NM, Claudin low)
Basal
Basal_NM
ClaudinLow
Luminal
PADI2
log2 gene expression level
15
10
5
0
−5
21MT1
21MT2
21NT
21PT
HCC1143
HCC1569
HCC1599
HCC1806
HCC1937
HCC3153
HCC70
JIMT1
MB157
MX1
SUM149PT
SUM229PE
184A1
184B5
MCF10A
MCF10F
MCF12A
BT549
HCC1395
HCC38
HS578T
MDAMB231
SUM1315MO2
600MPE
AU565
BT474
BT483
CAMA1
DU4475
EFM192A
EFM192B
EFM192C
HCC1419
HCC1428
HCC202
HCC2218
LY2
MCF7
MDAMB134VI
MDAMB361
MDAMB453
SKBR3
SUM190PT
SUM225CWN
SUM52PE
T47D
T47D_kBluc
UACC812
UACC893
ZR751
ZR7530
ZR75B
