# Supplementary material for: Identification of PADI2 as a potential breast cancer biomarker and therapeutic target
Source: BMC Cancer. 2012 Oct 30;12:500. doi: 10.1186/1471-2407-12-500 (PMC3571905; doi:10.1186/1471-2407-12-500)
Supplement: Additional file 4 — Figure S3. Flow-cytometry analysis of apoptosis in MCF10A and MCF10DCIS cell lines, and both proliferation/cell-growth and apoptosis in MDA-MB-231 cells. (A) MCF10A and MCF10DCIS cells were treated with different concentrations of Cl-amidine (0 μM, 200 μM, and 400 μM) and 10μg/mL Tunicamycin, and analyzed by flow-cytometry. Data represents percent apoptotic cells (cleaved Caspase-3 positive) after 2d and are expressed as the mean ± SD from three independent experiments (* p < 0.05, ** p < 0.001). (B) MDA-MB-231 cells were treated with increasing concentrations of Cl-amidine (0 μM, 100 μM, 200 μM, and 400 μM) over 4d and analyzed by flow-cytometry. Cell counts (DAPI) show a dose-dependent decrease in the growth (* p < 0.001). (C) Apoptosis levels (cleaved Caspase-3 positive) significantly increase over the control only after treatment with 400 μM of Cl-amidine. Tunicamycin (10μg/mL ) is shown as a control for apoptosis (* p < 0.01). Data are expressed as the mean ± SD from three independent experiments. [file 1471-2407-12-500-S4.pptx]

## Slide 1
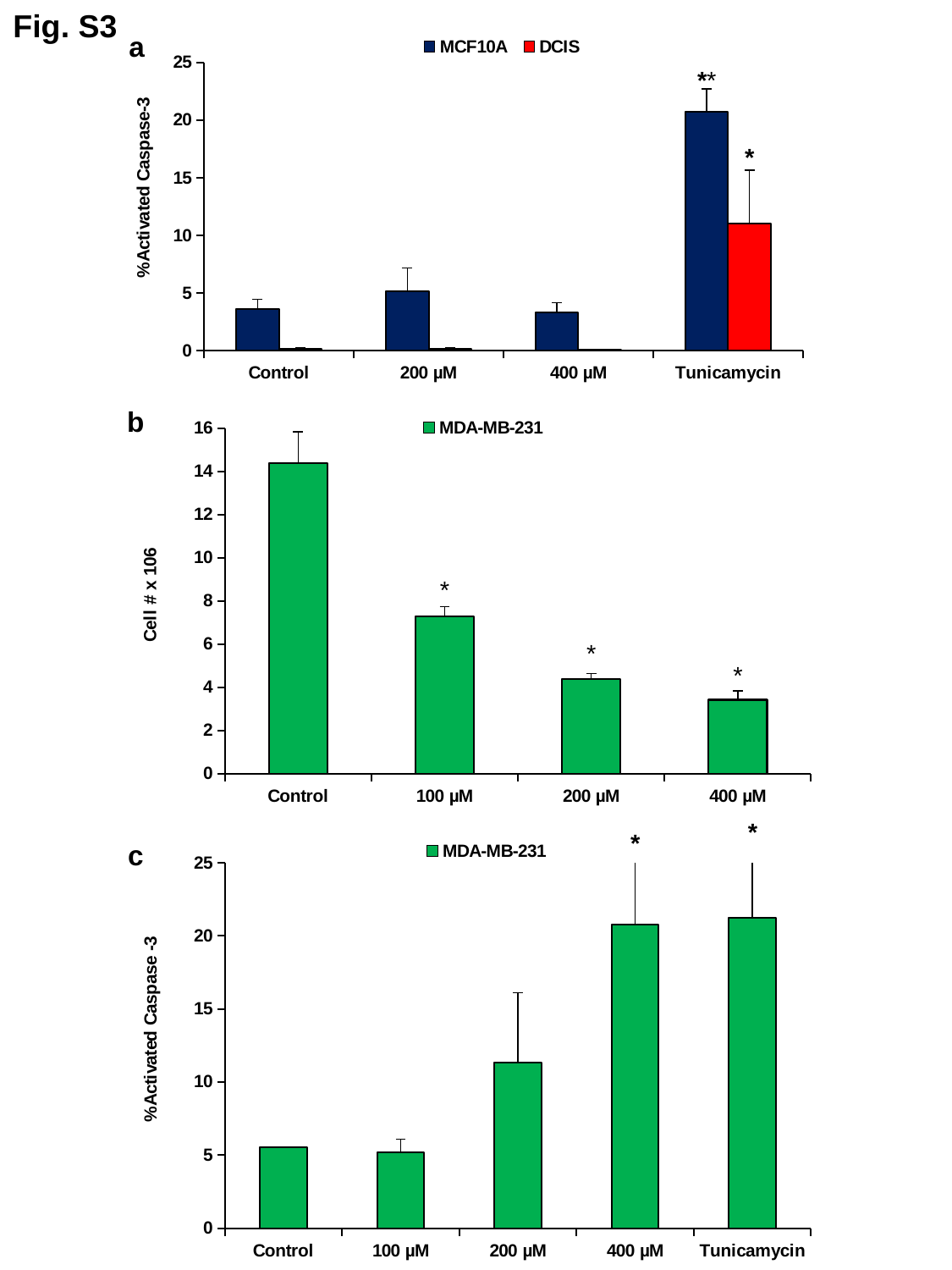

Fig. S3
### Chart
| Category | MCF10A | |
|---|---|---|
| Control | 3.6 | 0.16 |
| 200 µM | 5.17 | 0.2100000000000001 |
| 400 µM | 3.3 | 0.12000000000000002 |
| Tunicamycin | 20.71 | 11.06 |a
### Chart
| Category | |
|---|---|
| Control | 14.39 |
| 100 µM | 7.3 |
| 200 µM | 4.37 |
| 400 µM | 3.42 |b
### Chart
| Category | |
|---|---|
| Control | 5.55 |
| 100 µM | 5.1899999999999995 |
| 200 µM | 11.34 |
| 400 µM | 20.759999999999987 |
| Tunicamycin | 21.22 |c
